# Clinical Response to PI3K-α Inhibition in a Cohort of Children and Adults With *PIK3CA*-Related Overgrowth Spectrum Disorders

**DOI:** 10.1097/jova.0000000000000038

**Published:** 2022-03-02

**Authors:** Prashant Raghavendran, Sharon E. Albers, James D. Phillips, Sara Zarnegar-Lumley, Alexandra J. Borst

**Affiliations:** a Division of Pediatric Hematology-Oncology, Monroe Carell Jr. Children’s Hospital, Vanderbilt University Medical Center, Nashville, Tennessee;; b Department of Dermatology, Morsani College of Medicine, University of South Florida, Tampa, Florida;; c Division of Pediatric Otolaryngology—Head and Neck Surgery, Vanderbilt University Medical Center, Nashville, Tennessee;; d Division of Pediatric Hematology, Children’s Hospital of Philadelphia, Philadelphia, Pennsylvania.

**Keywords:** *PIK3CA*-related overgrowth spectrum, vascular malformation, alpelisib, therapy, Klippel Trenaunay

## Abstract

**Objective:**

The goal of this report is to describe, through a series of 5 cases, the clinical response and safety of alpelisib (BYL719) use in children and adults with *PIK3CA*-related overgrowth spectrum (PROS) disorders at our center.

**Methods:**

We reviewed clinical records of 5 patients from October 2019 through September 2021 followed by the pediatric hematology and multidisciplinary vascular anomalies teams at the Monroe Carell Jr. Children’s Hospital at Vanderbilt (MCJCHV). All patients carried a clinical or genetic diagnosis of PROS and were treated with alpelisib provided by a Novartis managed access program.

**Results:**

We highlight improvement in reported symptoms, objective overgrowth measurements, and quality of life to varying degrees in all patients. We note dose-dependent hyperglycemia and gastrointestinal side effects in 2 of the 5 patients. No patients experienced any serious side effects.

**Conclusion:**

This case series reports on the real-world use of PI3K-α inhibition in the management of PROS. Ongoing clinical trials will provide efficacy and safety data as these drugs become more widely used in patients with vascular anomalies and syndromes secondary to somatic *PIK3CA* mutations.

## Introduction

The *PIK3CA* (phosphatidylinositol-4,5-bisphosphate 3-kinase catalytic subunit alpha) gene encodes a series of lipid kinases (PI3 kinases) that play a critical role in cellular proliferation and survival via the PI3K-AKT-mTOR intracellular pathway.^1^ Mutations in *PIK3CA* cause a spectrum of somatic overgrowth and vascular disorders, now referred to as *PIK3CA*-related overgrowth spectrum disorders (PROS).^1,2^ In addition to severe overgrowth and vascular malformations, patients with PROS may experience orthopedic complications, endocrinopathies, thrombosis, multiorgan dysfunction, and airway compromise.^3,4^ Expanded awareness of PROS in the past decade has led to increased recognition of the disease state as well as a better understanding of the molecular etiology. The discovery of the link to the *PIK3CA* gene, which encodes the PI3K-α subunit and is known to facilitate tumor growth in malignancy, has provided a therapeutic target via PI3K-α inhibition.^5^ Venot et al^2^ first published clinical response to a PI3K-α inhibitor, alpelisib (BYL719), in 2018 in a retrospective series of 19 patients with PROS. All patients in that series experienced improvement in disease-related symptoms and there were no significant side effects noted. Expected toxicities based on prior available data from use in oncology include hyperglycemia, diarrhea, stomatitis, anemia, fatigue, and rash.^6,7^ Since this initial report, use of PI3K inhibition in PROS has been published in only a small number of case reports.^8,9^ Alpelisib, which selectively binds to and inhibits the p110α protein subunit, is currently being investigated in patients with PROS via the Phase II EPIK-PK Trial (NCT04589650).^10^ For patients unable to participate in the clinical trial, the drug is also available via a managed access program (MAP) through the manufacturer, Novartis. In this report, we detail the experience of 5 patients with PROS treated with alpelisib at our institution via a MAP. Because patients with PROS can have a highly variable clinical phenotype, response to PI3K inhibition may also vary depending on underlying mutation and expression, specific manifestations of disease, and comorbidities.^1,11^ The goal of this case series is to add to the growing body of literature on the varying presentations of PROS as well as reporting on individual patient response and safety.

## Methods

We reviewed clinical records of all patients at MCJCHV who enrolled in the Novartis MAP to provide alpelisib in PROS. Clinical history before enrollment in the MAP was abstracted from the electronic health record. We reviewed clinical and genetic diagnosis, reported side effects, dose and duration of alpelisib therapy, clinical photographs, exam measurements, and reported change in symptoms and quality of life. All patients over 18 years of age and parents of patients under age 18 years gave informed consent to access medical records for review as well as to document their cases and provide photographs for publication. The 2 patients under 18 years also provided assent. As part of the local IRB-approved protocol for patients treated on the MAP, patients were seen monthly for the first 6 months of their therapy and then at every 3-month intervals following that. Patients had an extensive baseline laboratory evaluation before alpelisib initiation (as recommended by the MAP) and then had complete blood count and comprehensive metabolic panel at each visit, with hemoglobin A1C monitoring every 3 months. All patients were initiated on a dose of 50 mg once daily with escalation only after established tolerance of the drug for at least 3 months and dose increase in 50 mg increments no more frequently than once per month. After our own experience and other reported data about increased adverse effects at doses >150 mg, no patients were further escalated above a 150 mg daily dose. Dose reduction back to previously tolerated dose was performed for any noted side effects.

## Results

A summary of each patient’s clinical and genetic diagnosis, duration of drug therapy, reported change in symptoms, and any adverse effects is summarized in Table 1.

### Case 1

**Figure 1.**
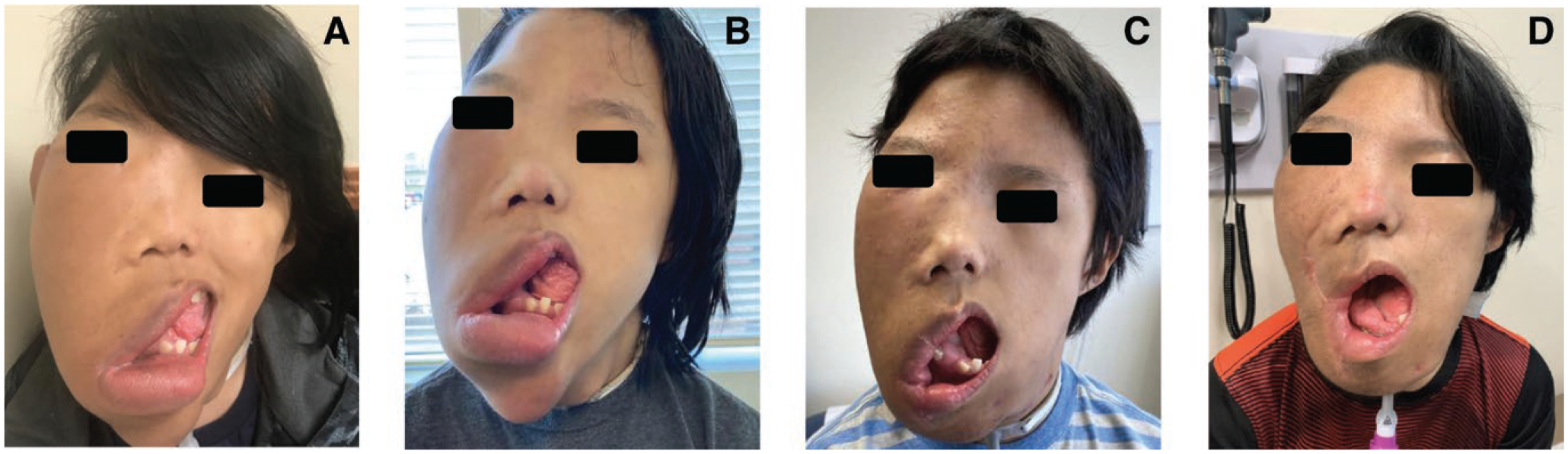
(A–D). Clinical photographs, case 1. Images demonstrate improving facial asymmetry in case 1 while on alpelisib therapy, shown at: (A) diagnosis, (B) 6 months of therapy (before any surgical procedures), (C) 12 months of therapy, and (D) 24 months of therapy. Patient did undergo facial debulking and lip reconstruction between panels (B) and (C) as well as panels (C) and (D).

A 14-year-old male born in China presented shortly after birth with cervicofacial capillary malformation, epidermal nevus, and bony and soft tissue overgrowth. He was orphaned in China and then adopted at age 3 years by a family in the United States. Early history and family history were unknown, but by age 3 years, he had significant overgrowth of the right face and bony facial structures, affecting hearing, speech, eye opening, and his airway. He required tracheostomy placement at age 6 years. He had several large debulking procedures, one of which resulted in right facial nerve paralysis. Cutaneous features included capillary malformation over the nasal bridge and epidermal nevus extending along the right lateral face in the preauricular region and down to the neck. This was partially removed in one of the debulking procedures. Apart from effects on hearing and speech, patient was neurologically normal with normal cognition and development. A biopsy from affected tissue confirmed an activating mutation in *PIK3CA* (c.3140A>G;p. His1047Arg), confirming a genotype of *PIK3CA H1047R*. This mutation is responsible for a gain of function in the kinase domain of the *PI3KCA* protein.^12^ It is implicated in CLOVES syndrome (congenital lipomatous overgrowth, vascular malformation, epidermal nevi, and scoliosis), macrodactyly, and fibroadenomatous overgrowth syndrome among others.^13^ The patient started alpelisib via Novartis MAP in October 2019 at a dose of 50 mg once daily that was increased to 100 mg once daily 11 months later. He has reported no side effects to date. He developed mild, self-resolving anemia following a few intercurrent surgical procedures and hyperglycemia labs. After starting alpelisib, the patient noted a marked reduction in soft tissue overgrowth of the right hemiface (Figure 1A, B). Imaging was not performed for monitoring of drug response, but monthly photographs were taken and showed improvement in bulk, which was also self-reported by the patient and family. Between 6 and 24 months following initiation of alpelisib, the patient had 3 total surgical procedures; complex otoplasty, a staged procedure to decrease bulk and improve closure of the lip, and right frontal bone reduction. Additional mandibular reduction is planned for the future. The medical and surgical teams felt that alpelisib prevented regrowth and recurrence between surgeries. Imaging performed as part of staged surgical procedures did reveal decreased bony and soft tissue bulk, but this was also affected by the surgical procedures performed.

### Case 2

A 42-year-old female presented with a diagnosis of Klippel-Trenaunay syndrome and capillary-venous-lymphatic malformation with overgrowth of the right lower extremity. She had an extensive history of deep vein thrombosis with pulmonary embolism, multiple prior sclerotherapy procedures, and chronic pain syndrome with opioid dependence. She had been on long-term anticoagulation and sirolimus for nearly 10 years. Due to persistent pain and gait alteration as well as dermatologic side effects from sirolimus, patient decided to initiate alpelisib therapy. She had 2 prior biopsies for genetic testing, but neither identified any pathogenic mutations in *PIK3CA* or other known genes associated with overgrowth (somatic overgrowth gene panels at both Washington University in St. Louis and University of Pennsylvania). Patient started on 50 mg daily but was increased to 250 mg daily within a period of 3 months per her request. She developed severe hyperglycemia requiring dose reduction to 150 mg daily accompanied by dietary modifications, which led to resolution of hyperglycemia within 4 months. Furthermore, within 4 months of starting alpelisib, patient self-discontinued chronic opioid medication and started a daily walking program. She Her leg circumference and foot size have markedly decreased from right thigh circumference of 53.5 cm to 48 cm now. Shoe size decreased from women’s size 8 to 6.5. She reports an enhanced quality of life due to significant improvement in pain, sleep, and mood.

### Case 3

A 42-year-old male presented to our clinic with a diagnosis of Klippel-Trenaunay syndrome. His phenotype was consistent with diffuse capillary malformation with overgrowth as well as venous malformation. He had significant right-sided overgrowth, including facial, nasal, chest, back, and extremities. His clinical history of obstructive sleep apnea and chronic nasal drainage were attributed to narrowed nasal passages from overgrowth. He had pain with prolonged walking/standing and edema in both lower extremities. Prominent venous markings were noted of the lower extremities and magnetic resonance imaging revealed venous malformation throughout the back and pelvis. He underwent biopsy of affected overgrown extremity with capillary malformation which revealed a *PIK3CA* mutation c.3139C>T;p. His1047Tyr, confirming a genotype of *PIK3CA H1047Y*, which confers a gain of function that causes increased activation of downstream signaling. This mutation has been reported previously in macrocephaly-capillary malformation.^13^ He decided to initiate therapy with alpelisib due to extremity size, pain, and need for improvement in nasal drainage/sleep issues. After initiating therapy at a dose of 50 mg daily, he noted some worsening of baseline irritable bowel syndrome symptoms. This stabilized after a couple of months, but after an increase to 100 mg daily, worsened again. Dose was decreased back to 50 mg daily, There were no hematologic abnormalities and no significant hyperglycemia on therapy.

### Case 4

A 19-year-old female presented to our clinic at age 16 years with diagnosis of Klippel-Trenaunay syndrome. She was born with extensive capillary malformation along the entire right side of her body, including some facial involvement, and right-sided hemihypertrophy. She had a normal ophthalmologic exam in childhood and was mostly asymptomatic until adolescence when she developed occasional right hip pain, right leg swelling, and aching with prolonged standing or walking. Family history was notable for unprovoked deep vein thrombosis in a first degree relative but no history of vascular anomalies. She has had no evidence of venous or lymphatic malformation on either clinical exam or imaging. Biopsy of affected skin showed *PIK3CA* mutation c.311C>T;p.Pro104Leu, confirming a genotype of *PIK3CA* P104L. This variant has been reported in PROS and Cowden syndrome and is categorized as likely pathogenic based on the disruptive nature of the missense mutation, but not confirmed by functional studies.^11^ She started on alpelisib at 50 mg once daily and increased to 150 mg once daily over a period of 6 months. She has not had any side effects or laboratory abnormalities to date. She has had marked reduction in pain with standing and walking and notable decreased bulk in her right upper and lower extremities.

### Case 5

**Figure 2.**
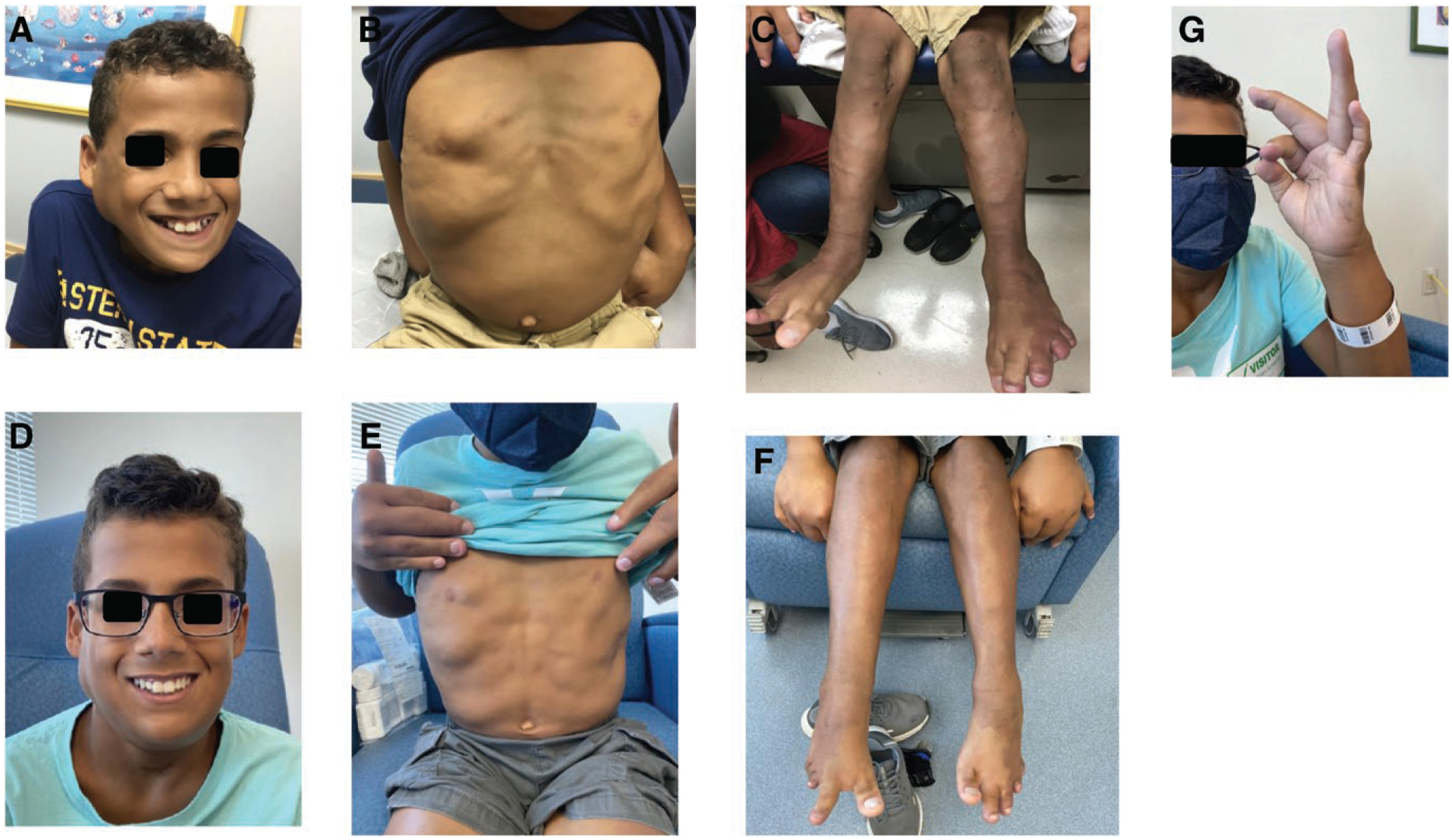
(A–G). Clinical photographs, case 5. Images comparing facial, torso, and lower extremity deformity at diagnosis (A–C) and 6 months into alpelisib therapy (D–F). Improved pincer grasp also demonstrated (G).

A 13-year-old male was diagnosed with CLOVES syndrome shortly after birth with significant overgrowth of hands and feet, multiple lymphatic malformations with recurrent infection, extensive venous malformation of the bilateral lower extremities, and capillary malformation with lymphatic blebs on the skin surface. Genetic testing for *PIK3CA* mutation was reported to have been done at a prior treating institution, but report was not available for confirmation or review. Repeat biopsy and genetic testing was planned but not covered by the patient’s insurance. Patient had multiple recurrent episodes of lymphatic malformation swelling, pain, and superinfection requiring frequent courses of antibiotics and hospital visits. At age 10 years, he started sirolimus therapy, with decrease in frequency of episodes, but continued pain and one significant episode of lymphatic malformation bleeding requiring hospitalization. He also continued to experience ongoing overgrowth of hands and feet, making writing, holding objects, and playing the piano difficult. After long discussion with family, decision was made to switch from sirolimus to alpelisib therapy. He had mild nausea and gastrointestinal upset when starting therapy at 50 mg daily, which resolved after switch from morning to evening dosing. He tolerated an increase in dose to 100 mg nightly without any additional side effects. He has had no laboratory concerns and no hyperglycemia. He has noted improvement in pain, no new flares of lymphatic malformations, and improvement in hand grip and dexterity since starting alpelisib 6 months ago. He has noted improvement in facial asymmetry, decreased venous distention in the lower extremities, and ability to perform pincer grasp, which was previously impossible (Figure 2).

## Discussion

The discovery of the molecular etiology of many vascular anomalies and overgrowth syndromes has heralded the possibility of a more targeted approach to medical therapy.^14^ mTOR inhibition with sirolimus has been demonstrated to be effective in reducing pain, lymphatic leak, and quality of life in many patients with vascular malformations,^15^ but has generally been less effective in treatment of overgrowth as seen in the majority of patient reports of PROS.^16^ The discovery and subsequent empiric use of PI3K inhibitors in this patient population provides a novel approach to therapy for patients with overgrowth as well as patients with limited response to or toxicity from mTOR inhibition.

In this series of 5 patients with PROS spectrum disorders, we report on clinical response to alpelisib therapy as well as safety and tolerability. All patients in our cohort reported some improvement in symptoms after starting the drug which contributed to their desire to continue therapy through the MAP. Patients 1 and 2 experienced the most dramatic improvement in symptoms, and patient 1’s response was more objectively apparent on examination than in photos. Patient 3 has had very minimal improvement in symptoms to date, but also remains on very low dose of the drug to due side effects at higher dosing. We had patient with dose limiting hyperglycemia and patients with gastrointestinal side effects. No patients have had any serious adverse events and none have required medical or surgical intervention for side effects nor discontinuation of drug.

The definition of PROS and clinical criteria for the umbrella definition were first agreed upon in 2015.^17^ Since that time, there has been increasing awareness of the definition as well as increased availability of testing for somatic activating mutations in *PIK3CA*. Improved understanding of the clinical spectrum of PROS as well as increased molecular testing and awareness will improve genotype-phenotype correlation. The availability of targeted agents, many adopted from the oncology world, has opened new therapeutic possibilities for patients with severe and life-threatening symptoms related to their disorder. Ongoing efforts through formal clinical trials will be crucial to defining medication safety and efficacy. As demonstrated by our cohort, alpelisib can result in appreciable symptomatic improvement for patients, but adverse effects in a subset of patients have also limited the ability to provide maximal dosing and accompanying benefit.

Limitations of this case series include a retrospective assessment of patients being treated outside of a clinical trial setting with no formal toxicity or quality of life measurements and lack of molecular confirmation in 2 of 5 patients. The medical team initiated therapy in these cases based on the determination of significant clinical need, lack of sufficient improvement in symptoms with sirolimus, and clinical phenotype highly consistent with PROS despite lack of molecular confirmation.

The implications of treatment response among various specific mutations within the *PIK3CA* gene are not yet fully understood in these disorders and much of what is currently known comes from the oncology literature.^18–20^ In patients with advanced cancers, the H1047R mutation (as seen in case 1) has shown greater response than other variants to PI3K inhibition.^19,21^ Partial response has been reported in patients with the P104L variant, which likely disrupts the association of p110α with the p85 subunit.^21^ Other *in vitro* studies have shown potential benefit for adjunct medications, such as thymoquinone, that can target H1047R and H1047L mutations, thus inhibiting the pathologic pathway.^18,20^ Little has been reported on the efficacy of alpelisib in patients harboring H1047Y mutations, but a large phase I study in adults with solid tumors suggested little response to PI3K inhibition in patients with this mutation.^22^ This is interesting, considering our patient from case 3 who experienced little benefit from the therapy. However, more data are needed about how genotypic variation within PROS spectrum disorders predicts outcomes with these newer therapeutic agents. Additional information is also needed to understand the effect of targeted drug therapy on other clinical features, such as developmental issues in megalencephaly-capillary malformation syndrome. Patient quality of life measures will also be key to determining therapeutic success. In addition, for patients with less severe disease manifestations, determining when and if to initiate targeted therapy will be an important conversation in the future for our field.

## Conclusion

Discovery of the importance of the PI3K-AKT-mTOR intracellular pathway in the etiology of *PIK3CA*-related overgrowth syndromes has led to increased recognition of a spectrum of clinical phenotypes and the possibility of targeted therapy through PI3K-α inhibition. Here, we report on the use of the PI3K-α inhibitor, alpelisib, in the clinical management of 5 patients with PROS. Our hope is that this report adds both additional information for genotype-phenotype correlation in PROS as well as provides some valuable safety and efficacy data on the use of PI3K-α inhibition outside a formal clinical trial setting.

## Figures and Tables

**Table 1.** Summary of Patient Cases

Patient	Phenotype	Genotype	Months to Dati on Alpelisib Therapy	Clinical Response	Adverse Events
1	PROS: hemifacial hypertrophy epidermal nevus, capillary malformation	*PIK3CA H1047R* (c.3140A>G;p.His1047Arg)	24	Marked improvement in facial overgrowth. Improvement in eye and lip closure. Lightening of capillary malformation.	None
2	Klippel-Trenaunay syndrome; capillary-venous-lymphatic malformation with overgrowth	None	15	Slight decrease in circumference of right lower extremity, decreased size of right foot (change in shoe size). Marked improvement in pain and exercise tolerance.	Hyperglycemia; resolved with dose reduction from 250 mg to 150mg daily and diet/exercise changes
3	PROS: capillary malformation with diffuse overgrowth (right side, including facial), venous malformation	*PIK3CA H1047Y* (c.3139C>T;p.His1047Tyr)	15	Slight decrease in right facial and right lower extremity overgrowth. Mild improvement in pain symptoms.	Increased diarrhea and fecal urgency
4	PROS: capillary malformation with diffuse overgrowth (right side, including facial)	*PIK3CA P104L* (c.311C>T;p.Pro104Leu)	9	Slight decrease in right upper and lower extremity overgrowth. Marked improvement in pain and gait.	None
5	CLOVES	Reported *PIK3CA* mutation, but report not available/confirmed by our center	6	Some improvement in lower extremity overgrowth. Improvement in pain. Improvement with hand dexterity and new ability to do pincer grasp and play piano.	Mild gastrointestinal upset; resolved with changing dosing from AM to PM

CLOVES, congenital lipomatosis, overgrowth, vascular malformations, epidermal nevi, spinal/skeletal anomalies or scoliosis; PROS, *PIK3CA*-related overgrowth spectrum.
